# Neurotransmitters Regulation and Food Intake: The Role of Dietary Sources in Neurotransmission

**DOI:** 10.3390/molecules28010210

**Published:** 2022-12-26

**Authors:** Amin Gasmi, Aniqa Nasreen, Alain Menzel, Asma Gasmi Benahmed, Lyudmila Pivina, Sàdaf Noor, Massimiliano Peana, Salvatore Chirumbolo, Geir Bjørklund

**Affiliations:** 1 Société Francophone de Nutrithérapie et de Nutrigénétique Appliquée, 69100 Villeurbanne, France; 2 Department of Physiology, King Edward Medical University, Lahore 54000, Pakistan; 3 Laboratoires Réunis, 38, Rue Hiehl, L-6131 Junglinster, Luxembourg; 4 Académie Internationale de Médecine Dentaire Intégrative, 75000 Paris, France; 5 Department of Neurology, Ophthalmology and Otolaryngology, Semey Medical University, 071400 Semey, Kazakhstan; 6 CONEM Kazakhstan Environmental Health and Safety Research Group, Semey Medical University, 071400 Semey, Kazakhstan; 7 Institute of Molecular Biology and Biotechnology, Bahauddin Zakariya University, Multan 60800, Pakistan; 8 Department of Chemical, Physical, Mathematical and Natural Sciences, University of Sassari, Via Vienna 2, 07100 Sassari, Italy; 9 Department of Neurosciences, Biomedicine and Movement Sciences, University of Verona, 37134 Verona, Italy; 10 CONEM Scientific Secretary, Strada Le Grazie 9, 37134 Verona, Italy; 11 Council for Nutritional and Environmental Medicine, Toften 24, 8610 Mo i Rana, Norway

**Keywords:** neurotransmitters, food, dietary supplements, precursors, health effects, nervous system

## Abstract

Neurotransmitters (NTs) are biologically active chemicals, which mediate the electrochemical transmission between neurons. NTs control numerous organic functions particularly crucial for life, including movement, emotional responses, and the physical ability to feel pleasure and pain. These molecules are synthesized from simple, very common precursors. Many types of NTs have both excitatory and inhibitory effects. Neurotransmitters’ imbalance can cause many diseases and disorders, such as Parkinson’s disease, depression, insomnia, increased anxiety, memory loss, etc. Natural food sources containing NTs and/or their precursors would be a potential option to help maintain the balance of NTs to prevent brain and psychiatric disorders. The level of NTs could be influenced, therefore, by targeting dietary habits and nutritional regimens. The progressive implementation of nutritional approaches in clinical practice has made it necessary to infer more about some of the nutritional NTs in neuropsychiatry. However, the importance of the intake of nutritional NTs requires further understanding, since there are no prior significant studies about their bioavailability, clinical significance, and effects on nerve cells. Interventional strategies supported by evidence should be encouraged.

## 1. Introduction

**Figure 1:**
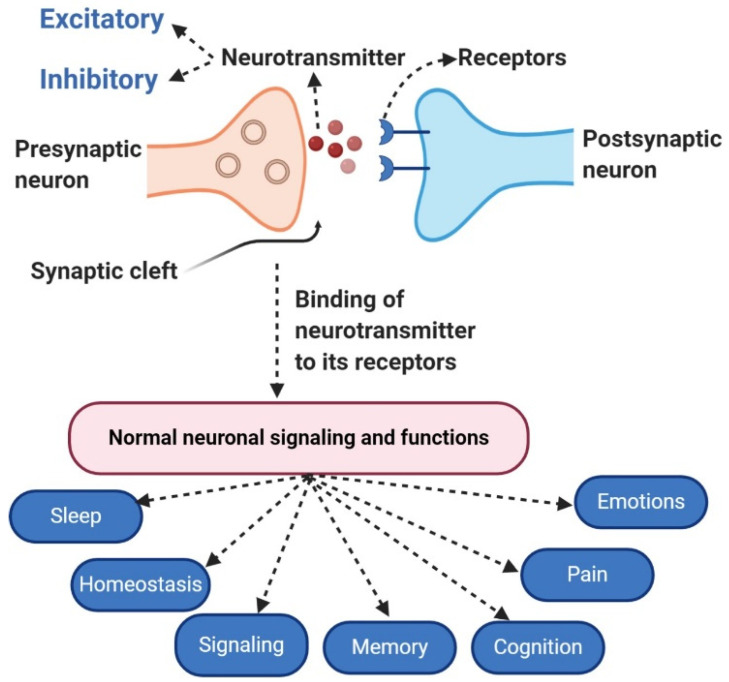
The diagram shows the basic mechanism of neurotransmission. NTs of both types (inhibitory and excitatory) are released from synaptic vesicles in synapses into the synaptic cleft. In the synaptic cleft, NTs are received by receptors present on the target cell. After binding with receptors, NTs provide signaling for various functions including sleep, homeostasis, pain, emotion, and cognition.

Neurotransmitters (NTs) are chemical messengers, which neurons use in the process of synaptic communication (neurotransmission) to interact with each other and with their target tissues [1]. NTs are produced to transmit information inside the brain as well as within the nervous system to other body parts. They are involved in the process of sensory information and motor-behavioral control. NTs are crucial for the complex functioning of the nervous system. The biosynthesis of many NTs involves simple and abundant precursors including widely available amino acids with few conversion steps. NTs synthesis takes place in nerve endings from where they are released and distributed into the synaptic cleft. From there, NTs reach the cell membrane of the target tissue, binding to receptor proteins. After the binding of NTs, the target tissue becomes activated, disrupted, or functionally modified. NTs are secreted by neurons, in a process that has been recognized as “action potential”: a condition that occurs due to an unbalanced ion’s charge. These chemical messengers can cross neurons over the synapse and bind to a receptor. From there, the message continues down the line to other neurons, or, if it has reached its endpoint, activates a distinct cell type to perform certain actions, depending on the NT and cell type. Some NTs act as receptor or pre-receptor signaling molecules, while others act at a post-preceptorial level [2,3]. NTs can have excitatory or inhibitory actions and some of them exhibit both functions based on the different situations. An excitatory NT causes alterations in the target cell while inhibitory NTs block any possible changes that can occur [4]. Figure 1 summarizes these simple concepts, illustrating the binding of NTs to the receptor and the activation of signaling of different functions.

**Figure 2:**
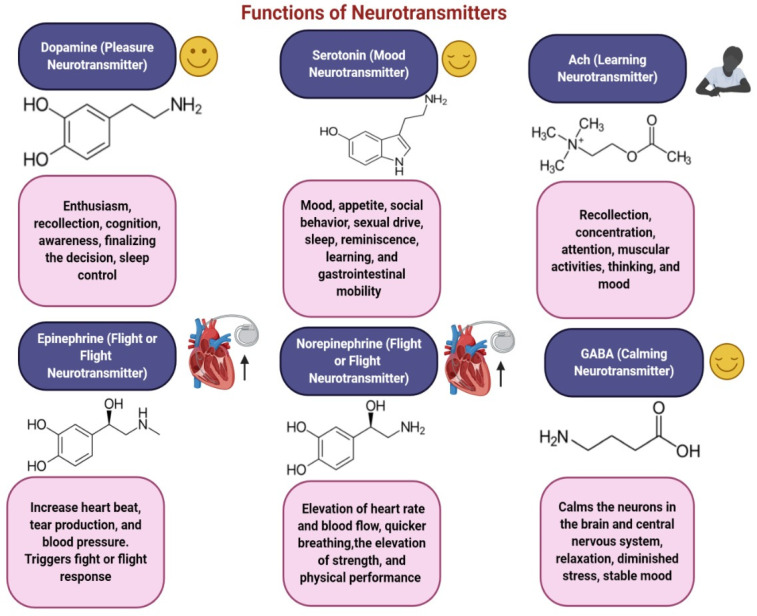
Illustration of major NTs along with their functions and chemical structures.

Most NTs, including dopamine (DA), gamma-aminobutyric acid (GABA), serotonin, and endocannabinoids, are synthesized within the gut and in the brain. About 95 % of the body’s serotonin comes from the gut, where it behaves both as a paracrine messenger and as a NT [5,6]. NTs are major functional molecules in the immune system, signaling the occurrence of certain actions [7]. The functions of some major NTs are shown in Figure 2. Studies have highlighted a possible linkage between NT dysfunction and many neurological and psychiatric disorders such as Parkinson’s disease, Alzheimer’s disease [8], depression, schizophrenia, borderline personality disorder [9], and fibromyalgia [10].

**Figure 3:**
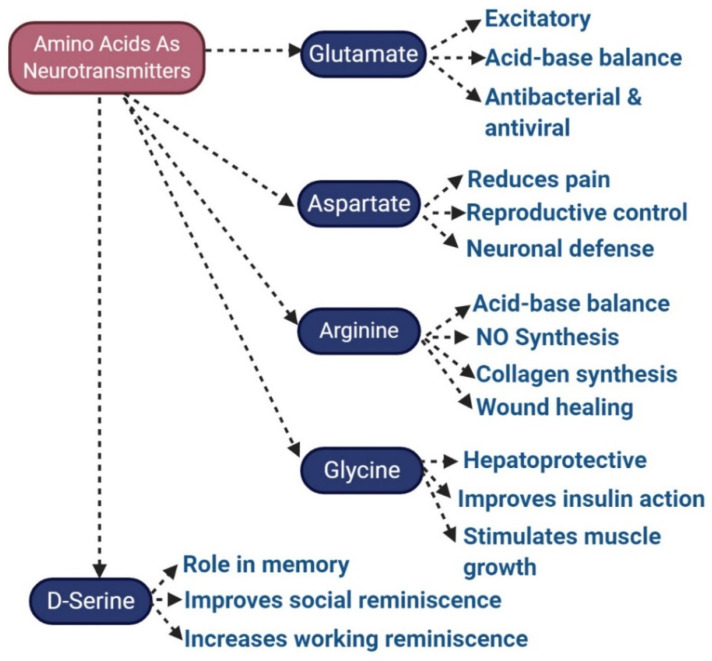
NTs based on amino acids and their major functions.

The precise number of distinct human NTs is still unidentified, but more than 200 NTs were identified and classified. The main types of NTs, depending on chemical and molecular characteristics, include amino acids like glutamate (Glu), aspartate (Asp), D-serine (D-Ser), GABA, glycine (Gly) (Figure 3); monoamines (including DA), norepinephrine (NE, which is also known as noradrenaline NA), epinephrine (EPI or adrenaline), histamine, serotonin, peptides like somatostatin (SST), substance P and opioids, purines, such as adenosine triphosphate and adenosine. Some gaseous substances, like nitric oxide, can also behave as an NT, and similarly, endogenous substances, chemically similar to monoamines, called trace amines, such as tryptamine and phenethylamines, can behave as NTs as well.

Acetylcholine (ACh), synthesized from neurons, is the parasympathetic nervous system’s primary NT, which regulates smooth muscle contraction and vasodilation, slowing the heart rate. GABA is the major inhibitory NT of the nervous system, which acts to attenuate the activity of the neurons. Defects in the release and function of NTs have been associated with numerous diseases and disorders, especially neuropsychiatric and neurodegenerative diseases. For instance, dysfunction of DA, Glu, and GABA NTs is already reported in the case of schizophrenia, while declines in norepinephrine and serotonin levels and activity have been observed in people with depression. A central feature of Parkinson’s disease is reduced DA levels, related to the loss of so-called dopaminergic neurons [11]. Although many drugs and medicines are present to maintain the regulation of NTs in the case of mental illness and disorders, however, these may pose some side effects [12]. 

## 2. Search Methods

**Figure 4:**
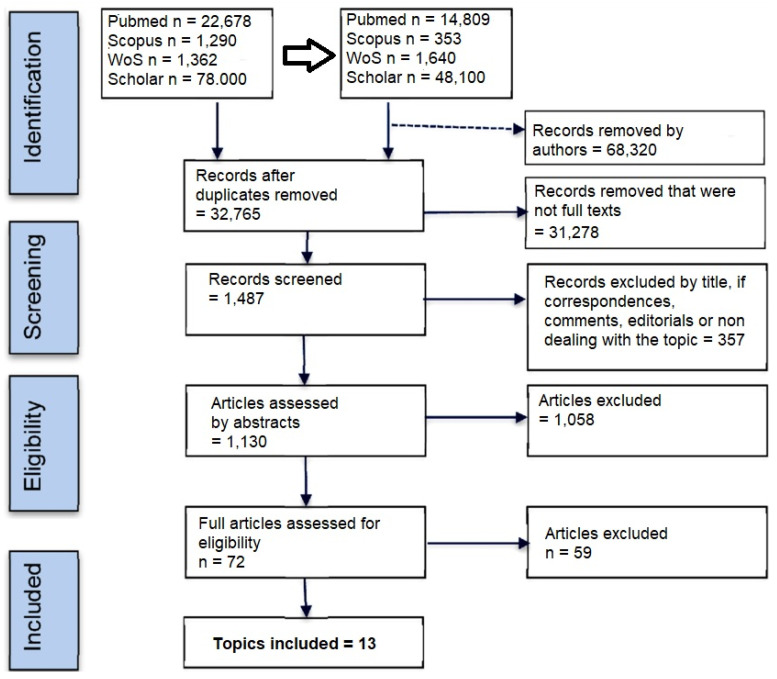
PRISMA Flow diagram of literature selection.

The literature search was performed by collecting all records matching the binary MESH terms “neurotransmitters AND diet” or “neurotransmitters AND nutrition”, on Pubmed, Scopus, Web of Science (WoS), and Google Scholar, retrieving several papers indicated in the Preferred Reporting Items for Systematic Reviews and Meta-Analysis (PRISMA) panel (Figure 4). 

Eligibility criteria were established before every paper selection, performed by two authors (GB and SC), on the basis of those papers a) written in English; b) published in impact and indexed journals; c) dealing with the role of diet and nutrition on the chemistry of NTs and/or their functions; d) preferentially with a clinical view. Exclusion criteria were considered if papers were in languages other than English, non-closely pertinent to the topic or non-full-length papers. Cohen’s k was 91.37% in accordance (k = 0.70941). 

A fundamental review of the role of diet in NTs functionality was reported by Briguglio et al. [13]. In this extensive review, the authors listed any nutrient and food source able to impact the NTs physiology and chemistry [13]. Richard Wurtman edited the chapter “Effects of Nutrients on Neurotransmitter Release” in 1994 [14], a research that was continued by others more recently [15,16]. Several foods naturally contain NTs. These food sources as dietary NTs can act as promising alternatives for improving nervous system-related disorders [17]. 

Food intake, diet changes, and nutritional supplements are known as outstanding options for complementary and alternative medicine, as they can either support or substitute traditional therapies. Current areas of medicine, like neuroscience and psychiatry, show a growing inclusion of alternative medicines in the modern therapeutic panorama. Health care practitioners continue to incorporate dietary changes with beneficial outcomes on some medical disorders, such as headaches [18]. These alternative medicines are often misused by patients beyond their therapeutic purpose [19], thus actually causing side effects, drug reactions, and waste of time and money [20]. The high intakes of alternative medicines in neuropsychiatric patients are largely consistent with the perception that food could be a modulator of mood. Food has indeed been identified to affect mood based on the availability of precursors for NTs and mechanisms of recompensing [21,22]. In addition, nutritional supplements of some micronutrients could help patients to recover from restlessness and depressive symptoms, with the integration of iron and folate, respectively [23]. Despite the growing interest in the therapeutic impact of food on the nervous system, inadequate details are available on food sources for NTs. ACh, Glu, GABA, and biogenic amines like DA, serotonin, and His, are equally present in some animal foods, fruits, edible plants, and roots [24]. There are similar chemical compositions of NTs present in both plants and animals. ACh, GABA, and serotonin can naturally occur either as primary or secondary metabolic products; they take part in important metabolic pathways [25].

Similarly, some trace amines are also present in diverse varieties of food, like synephrine and octopamine present in *Citrus aurantium* fruit [26], and anandamide in chocolate [27]. There are many essential nutrients for NT synthesis and regulation; especially amino acids such as the tryptophan (Trp) and tyrosine (Tyr) precursors, choline, B-vitamins (particularly vitamin B6, vitamin B12, and folate) vitamin C, valine, leucine, isoleucine, phenylalanine, zinc, iron, omega-3 fatty acids, and vitamin D. There are several types of food recognized for their positive effects on the brain function. Tea is one of the examples, most probably due to theanine that in the brain increases the levels of serotonin, dopamine and GABA [28,29].

Each NT has its own assigned substrate, including many of the aforementioned nutrients, which should generally be included in the diet. This review discusses nine of the major NTs, their nutritional associations, which foods should be consumed for an effective intake of each NT, and how their levels and substrates can be affected by diet.

## 3. The Relationship between Neurotransmitter Regulation and Food Consumption

### 3.1. Dopamine

Dopamine is a substantial modulatory NT within the brain. Dopamine cell bodies are present only in small clusters of nuclei within the “midbrain”, like the ventral tegmental area and substantia nigra. Dopamine can affect several areas of the brain in a systematic manner, modulating the way of thinking, feeling, and action [30]. It is synthesized by the aromatic enzyme L-amino acid decarboxylase, also termed DOPA decarboxylase, using the precursor L-Dopa. The DOPA decarboxylase enzyme is also used for serotonin and His synthesis. L-Dopa is indeed produced from the amino acid L-Tyr (from the Tyr hydroxylase enzyme) a process in which several other supporting chemical substances (cofactors) are required including tetrahydrobiopterin and iron. Tetrahydrobiopterin is also needed for the synthesis of many other NTs [31]. The precursor Tyr is mandatory for the synthesis of DA. Some shared cofactors are required to change Tyr into DA; these specifically are B complex vitamins and trace elements such as copper, zinc, or iron [32,33]. L-phenylalanine is another amino acid that can also synthesize L-Tyr, which is derived from the diet. Dopamine cannot readily cross the blood–brain barrier itself, and therefore must be synthesized within the brain tissue. Because DA works as a neurochemical modulator throughout multiple brain regions, it has the potential to affect many aspects of brain activity. There are many hubs of DA in the brain; the major ones are the ventral tegmental region that projects towards the nucleus accumbens and the prefrontal cortex alongside the substantia nigra region, which makes up part of the basal ganglia [34]. Each hub controls quite different functions within the brain. The function of the substantia nigra, for example, could be ascertained via the emotional, cognitive, and movement abnormalities shown by persons with Parkinson’s disease owing to the depletion of DA release from this hub. In the brain, one of the main functions of DA is reward learning and prediction, the process through which people adjust their behavior depending on assumptions of where and when rewards (including money, pleasure, food, or success) could perhaps occur in the future [35]. Deficiency of DA combined with substantia nigra degradation contributes to the condition of Parkinson’s disorder. Higher dopaminergic neuronal activation leads to the pathophysiology of psychiatric disorders and schizophrenia [36]. Drug and alcohol addiction could temporarily increase blood DA levels, leading to confusion and a loss of concentration [37]. Nevertheless, adequate DA secretion in the bloodstream plays an important role in motivating or seeking to complete the task. Many significant nervous system disorders are associated with DA dysfunctions. It has been shown that schizophrenia, which is a serious mental illness, involves excessive levels of DA in the frontal lobes, leading to psychotic episodes in these patients [38]. The substantia nigra destruction contributes to the disruption of the main supply of DA in the central nervous system [39]. Eating fish, eggs, and spirulina increases DA levels, which improves sleep, attention, mood, and memory [40,41].

As DA absorbed from food cannot cross the blood–brain barrier so cannot act on the brain, there are some plants, however, which produce L-DOPA, the metabolic precursor of DA. Maximum concentrations were observed in the *Mucuna* genus plant leaves and bean pods, particularly in *Mucuna pruriens* (velvet beans), which were used as a drug source for L-DOPA [42]. Another plant that produces large amounts of L-DOPA is *Vicia faba*, the plant that makes fava beans (or “broad beans”). Even so, the L-DOPA level in the beans is slightly lower than in pod shells and other plant parts [43]. *Cassia* and *Bauhinia* seeds also have significant amounts of L-DOPA [44].

There are foods that increase DA through providing L-DOPA. These foods specifically are animal products like meat, poultry, fish, eggs, milk products, chocolate, fruits and vegetables such as different kinds of apples, avocados, bananas, watermelon, tomato, spinach, pea, some beets, green vegetables, sea vegetables, legumes, and peanuts, oats, oregano, rosemary, particularly sesame and pumpkin seeds, soy products, turmeric, some types of wheat, and olive oil [45,46]. Studies have shown results on DA concentration in different fruits: banana peel (700 μg/g), banana pulp (8 μg/g), and an avocado (4–5 μg/g) [47]. Based on a small study, it was suggested that ingesting 250 g of cooked velvet beans considerably elevated DA levels, and it also lessened Parkinson’s symptoms when eaten one to two hours after a meal [13]. 

The production of DA is regulated by an amino acid: Try which plays a fundamental role in its formation. Therefore, having adequate levels of this amino acid is very important: it is found in all protein foods such as turkey, eggs, legumes (a lot of soy), dairy products, and beef. Among these foods, currently of considerable interest are beans that have high quantities of L-DOPA [48], the precursor molecule of DA. Several studies have shown that the daily intake of Try can ensure a surge in DA levels by promoting deep thinking and improving memory. An excess of saturated fats present in animal fats, butter, palm, and coconut oil can interfere with the uptake of DA in the brain. Recently, some researchers investigated the impact of protein restriction on nigrostriatal dopamine pathway using fast-scan cyclic voltammetry in laboratory animals (rat brain slices). The level of DA increased in the nucleus accumbens of adult animals upon protein restriction and in response to low and high-frequency trains of stimulation. On the contrary, during youth DA was reduced in the same experiment [49].

Dietary regimen can affect the DA physiology, for example, a prolonged high-fat diet can induce alterations in the DA reuptake, independently from the level and the expression of the DA transporter [50]. Therefore, different dietary habits can affect dopamine biology.

### 3.2. Acetylcholine (ACh)

Acetylcholine is the main NT in the brain; it plays a role in improving attention, understanding, and memory. ACh cells are found inside the brainstem and midbrain nuclei collections such as nucleus basalis, septum, substantia innominata, Broca diagonal band, pedunculopontine nucleus, and laterodorsal tegmental region. The ACh cells spread from here into nearly any area of the brain. In the brain, ACh is synthesized from the choline acetyltransferase enzyme by utilizing two chemical compounds, i.e., choline and Acetyl-CoA. First, the enzyme separates the acetyl portion from the Acetyl-CoA and then adds choline to generate the ACh [51]. ACh functions both within the brain, where it is involved in cognitive processes such as concentration, learning, and memory, and within the peripheral nervous system where it is a vital signaling chemical at the junction between nerves and muscles. This is also present in sensory nerves and the autonomic nervous system and has a role in playing the management of the “dream state” when a person sleeps instantly [52]. ACh plays a crucial role in normal muscle functioning. For instance, toxic plants such as curare and hemlock induce muscle paralysis by blocking sites of ACh receptor muscle cells. The well-known toxin botulin works by preventing vesicles in the node bouton from releasing ACh, resulting in the effector muscle being paralyzed [53]. Alzheimer’s disease is a neurodegenerative condition characterized by impairments in learning and memory. It is due to a deficiency of ACh in some brain regions. Myasthenia gravis is a rare chronic autoimmune disease defined by dysfunction of the ACh synaptic transmission at neuromuscular junctions, resulting in tiredness and muscle weakness without atrophy. Very commonly, myasthenia gravis arises from circulating antibodies at the postsynaptic neuromuscular junction that inhibits choline-to reach the ACh receptors, which suppress the excitatory effects of ACh at neuromuscular junctions on nicotinic receptors [54]. In a more unusual case, muscle weakness might occur due to a genetic defect in inherited parts of the neuromuscular junction, as opposed to developing via passive transmission from the immune system of the mother at birth or by autoimmunity in later life [55]. ACh is a significant neurotransmitter typically involved in brain activity, and its decreased amount has been connected with neurological dysfunction. Clinical research suggested that reduced levels of ACh in older persons result in delirium after an operation, noteworthy fluctuations in consciousness, distraction, and disordered thinking [56]. ACh is crucial for the nervous system and also for muscles. As it is synthesized from the simple compound choline, that act as an important precursor for ACh synthesis, as already stated, it is therefore helpful to eat choline-rich foods. To raise ACh levels it is necessary to eat more egg yolks, Brussels sprouts, and dairy products [57].

There are plenty of foods in which choline is present. According to the United States Department of Agriculture Research Service Food Composition Databases, foods containing ACh are egg yolks, turkey, beef, green split peas, liver, salmon, soybeans, mung beans, and lentils. According to the research, maximum concentrations of ACh are found in the nettle species of *Urtica dioica* L. and of *Urtica aureus* L. These species contain approximately 0.5 μmol/g dry weight of roots. Similarly, the fruits of *Citrus aurantium* L. (orange), *Fragaria vesca* L. (strawberry), and the *Raphanus raphanistrum* subspecies *sativus* L. (reddish) also contain ACh [58]. Mistletoe has traditionally been used in treating patients with high blood pressure, arteriosclerosis, and headache with hypertension, epilepsy, chorea, hysteria, and other neurological conditions. Mistletoe’s cardiac depressant and sedative effects were due to different biologically active elements, such as ACh itself, but also due to the presence of His and GABA.

However, the different levels of choline in the blood and biological fluids are also fundamental biomarkers of cardiovascular disease, but different dietary regimens enriched in choline should not be misled with early predictions of cardiac events [59,60].

### 3.3. Serotonin

Serotonin is an important monoamine NT within the brain. It is secreted from brainstem neurons and nerves that internalize the gastrointestinal (enteric nervous system) tract. Moreover, serotonin is present in platelets, which produce it during clotting (hemostasis). In the brain, the key serotonin hub is the raphe nucleus, but other regions, such as the linear caudal nucleus, oral nucleus pontis centralis, and the postrema region, produce 5-hydroxytryptamine (5-HT). Every group of cell bodies has a distinct connectivity pattern inside the brain [61]. Synthesis of serotonin depends on the bioavailability of its precursor, the amino acid L-Trp, which via the action of 5-Hydroxytryptophan, is converted into serotonin, in a metabolic process involving two enzymes, Trp hydroxylase, and amino acid decarboxylase [62]. Serotonin cannot cross the blood–brain barrier, but in some cases, its counterpart Trp can be transmitted if it is present in sufficient quantities relative to other amino acids that challenge the blood–brain barrier for entering the brain. Serotonin has a modulatory function like DA, which exerts its effect through several different brain areas [63]. Therefore, it has no particular function but instead “adjusts,” the brain’s behavior via a wide spectrum of neurological, mental, biochemical, and metabolic processes to better control them. This encompasses mood, wakefulness and sleep, appetite, aggression frequency, circadian rhythms, body temperature, and neuroendocrine activity. Insufficient serotonin production can result in decreased function of the immune system, as well as a variety of emotional disorders such as depression, problems with anger management, obsessive compulsive disorder, and even suicidal thoughts [64].

Serotonin is the major NT which is called “the happy molecule.” It plays a significant part in the regulation of mood, appetite, social behavior, sexual drive, sleep, reminiscence, learning, and gastrointestinal mobility [13]. Serotonin plays a significant role in brain function, primarily memory, and learning. Low serotonin levels can lead to sleep disturbances, depression, and headaches. Consumption of food such as kiwi, oranges, bacon, walnuts, and turkey help to increase serotonin levels. There are three chief sources of serotonin: fruits, vegetables, and seeds. They include bananas, cherries, chicory, cabbage, coffee beans, brinjal, green grapes, legumes, onion, hickory peanuts, hot peppers, kiwi, green lettuce, oats, papaya, green pears, pineapples, plums potatoes, green spinach, strawberries, tomatoes, and walnuts [65].

According to recent research, the accretion of serotonin was equally perceived in *Capsicum annuum*, and paprika [66,67]. Similarly, serotonin was recognized in *Corylus avellana* L. (hazelnut), fruits of tomato and cherry tomato, *Ananas comosus* L. (pineapple), *Prunus domestica* L. (plum), *Carica papaya* L., and fruits of the *Actinidia genus* (kiwi) [66,68,69,70]. Comparable to DA, serotonin was found in the velvet bean [71]. *Griffonia* was used for its anxiolytic effects, which were mainly related to the amount of 5-hydroxyl-tryptophan, a specific precursor involved in the production of serotonin [72].

There are numerous varieties of dietary nutrients that increase serotonin levels: these are amino acids, vitamins, minerals, herbs, and many more. Typically, many cofactors are needed to create an effective serotonin supplement, in addition, also there can be some adverse reactions with medications. Therefore, precautions should be taken before using these supplements. These supplements are fundamentally amino acids. Trp, the precursor of serotonin, is an indispensable amino acid that could be found in numerous foods. Based on research, it was suggested that Trp supplements could increase brain serotonin [73]. Some plants have been found to contain serotonin, for example, nettle and *Griffonia simplicifolia. Griffonia* is used for its potential anxiolytic effects that are related to the production of 5-hydroxy-l-tryptophan, a possible precursor in serotonin synthesis [74].

The dietary consumption of amino acids may affect the serotonin-mediated modulation of stress and mood-related affective disorders [75], and the effect of diet on the serotoninergic neurotransmission in depression may be relevant [76].

### 3.4. Gamma-Aminobutyric Acid (GABA)

Gamma-aminobutyric acid (GABA) is the most important inhibitory NT in the human brain cortex. GABA is an amino acid synthesized in the brain. It plays an essential role as a NT, and one of its functions is to ease up communication between the cells of the brain. There are several roles of GABA: a decrease in the activity of the neurons in the brain and central nervous system, relaxation, diminished stress, stabilization of the mood, decreasing of pain, and sleep improvement [77,78]. 

GABA ensures that the brain does not transmit impulses “too quickly,” helping to keep the average neuronal activity level in balance for the brain. GABA is synthesized by the enzyme glutamic acid decarboxylase (GAD) from Glu, the principal excitatory neurotransmitter throughout the brain. The synthesis of GABA also involves a substantiating compound, a cofactor known as pyridoxal phosphate, obtained from the dietary vitamin B6. When GABA levels increase in the brain, GABA inhibits GAD’s action, then controlling its synthesis rate. GABA is not only released from inhibitory cells, but also from the supporting brain cells “glia”, and it is often “co-released” along with other NTs [79]. The GABA release pathway within the brain is further complicated by the fact that it can be activated from both ends, i.e., the axons and the dendrites of a brain cell. Multiple GABA release modes help ensure that its response can be dynamically fine-tuned to match the neural environment that is occurring. Again, when not needed, GABA has difficulty crossing the blood–brain barrier; thus, there is strong regulation of GABA levels in the brain. GABA is involved in a wide range of operations intended to finely tune neural processing. It is also commonly responsible for supporting sleep, for example, by the inhibition of wake-promoting areas. Interruption in GABA signaling is one significant contributor to anxiety disorders that could be treated with benzodiazepines which act to increase GABA signaling in the brain and thus eliminate negative brain excitability [80,81]. Some epileptic problems are caused due to the deficiency of inhibitory NTs like GABA, Medication meant to increase GABA can be used, depending on the trigger of the seizures [82]. A severe decline of GABA in the brain may result in Huntington’s disease, in addition to epilepsy. Although this is a hereditary condition due to DNA abnormality, one of the consequences of this disordered DNA is the neurons’ decreased capacity to pick up GABA. Huntington’s disease cannot be cured but symptoms can be controlled by therapeutically increasing the number of inhibitory NTs [83].

GABA is well known for its analgesic, anti-depressive, and hypotensive activity. Food technology and molecular engineering are used to synthesize GABA via enzymatic or whole-cell bio-catalysis, microbial fermentation (such as GABA soy yogurt, and black raspberry juice), and chemical synthesis [84,85]. Some authors found that one of the highest dry weight contents on GABA was 414 nmol/g in raw spinach, followed by *Solanum tuberosum* L. (potato), *Ipomoea batatas* L. (sweet potato), and *Brassica oleracea* L. (cruciferous). *Lentinula edodes* B. (Shiitake), i.e., mushrooms and nuts of the genus *Castanea* (chestnut) also showed the presence of large quantities of GABA. The highest concentration of GABA in white tea was observed among several varieties of Chinese teas [86]. GABA content, as already described, was present in mistletoe but also found in *Phytolacca americana* L., *Valeriana officinalis* L., *Hypericum perforatum* L., *Angelica archangelica* L., *Hieracium pilosella* L., and *Passiflora incarnata* L. *Passiflora incarnata* L. has been used to relieve mild mental stress symptoms and also as a sleep aid [87]. According to one study, the intake of a beverage containing GABA decreased symptoms of fatigue and stress. Other research showed that eating 10 g of chocolate along with 28 mg GABA ameliorated the stress reaction during a task and contributed to a more rapid recovery from a depressed state to a happy state [88].

Based on a study, it was demonstrated that the primary precursor for GABA production is glucose, which is degraded to Glu by the tricarboxylic acid cycle enzymes, via pyruvate and glutamine can equally act as precursors. The enzyme glutamic acid decarboxylase, found nearly solely in GABAergic neurons, catalyzes the alteration of glutamate to GABA. Similarly, GAD requires a cofactor, pyridoxal phosphate, for action [89].

Dried fruit also favors the synthesis of GABA, as walnuts, almonds, and hazelnuts are rich in glutamic acid. They also ensure essential fatty acids that are involved in the production of GABA. Glutamic acid is then contained in legumes (lupins, broad beans, broad beans, lentils). These foods also provide vitamin B and sugars which facilitate their production by the brain» adds the expert. The precursor of GABA is also present in spinach, tomatoes, and parsley. Eggs, fish, and meat are also excellent sources. These foods are also rich in Trp, an amino acid precursor of serotonin, a neurotransmitter which in turn helps regulate its production» continues the expert. Glutamic acid is used as a flavor enhancer in the food industry in the form of sodium, potassium, calcium, and magnesium glutamate. It is an additive present in ready meals such as soups, frozen foods, sauces, cured meats, stock cubes, used as a flavor enhancer. It is used to make these foods particularly tasty. On product packaging, it is indicated with the abbreviation E followed by a number (for example E620, E625). Excessive consumption of glutamate in the diet may alter the fine balance between glutamate and GABA [90].

### 3.5. Glutamate

Glu is one of the human brain’s most plentiful amino acids and has an excitatory action. This indicates that it contributes to the “activation” of a cell when it attaches to complementary receptor sites on the target neuron cell. When individuals get too much Glu in the brain, it could lead to destruction of the brain cells (it is poisonous at high concentrations) and thus Glu levels need to be tightly monitored to guarantee that the brain is not “overstimulated.” Glu is synthesized by the glutamine molecule which is another amino acid generated from the degradation of Glu by the glutaminase enzyme [91]. Since so much Glu is harmful to the body, it is typically kept locked up within the brain cells and released only when needed. Furthermore, when it is not required, Glu does not easily pass through the blood–brain barrier, which requires more regulation to ensure that the levels of Glu in the brain do not get too high. Assuming that Glu is the most stimulating NT in the brain, it is present to a certain degree throughout nearly all brain areas. It also plays a particular role within a neural mechanism known as synaptic plasticity, crucial for our learning process. This is how it can enhance or disrupt individual synapses, i.e., increase the strength of the subsequent signals transmitted from the synapse onwards. While doing so, synaptic plasticity changes and improves communication patterns of the brain to consider new information learned that should be stored in memory, basically underpinning the idea of possessing a “plastic” brain [92,93]. Many epileptic disorders are induced by the rise in excitatory NTs like Glu; treatment is aimed at decreasing Glu according to the cause of the seizures [94].

Glu is universal and found in nearly all living organisms. It is the prime stimulatory NT found in the central nervous system. Glutamic acid is naturally found in foods with a high protein amount. These foods are meats, seafood, stews, soups, and sauces. Similarly, seaweeds, cheeses, fish sauces, soy sauces, fermented beans, and *Solanum Lycopersicum* L. (tomato) presented more significant amounts of free glutamic acid. The other well-known sources of this amino acid are dried cod, cracklings, salami, caviar, and instant coffee powder, salts of glutamic acid, like sodium, potassium, calcium, and magnesium, which can be added to some foods or sauces as flavor enhancers [95]. Whenever these foods are eaten, monosodium glutamate and other glutamate salts separate, forming Glu. In the same manner, food sources of monosodium glutamate and glutamic acid are typically the same: fish sauces, oyster sauce, tomato sauce, gravies, miso, noodles dishes, Parmesan cheese, savory snacks, chips, ready-to-eat meals, but likewise, mushrooms and spinach [96].

Consequently, meat and other animal products are the weakest sources to obtain high quantities of glutamate. Lysine is a substantial precursor for the de novo manufacture of Glu. An extensive study demonstrated that the creation of glutamate from lysine occurs through the saccharopine pathway in neurons. Similarly, ornithine is also a precursor of the neurotransmitter Glu [97].

### 3.6. Norepinephrine

NE, sometimes called noradrenaline, is a particular catecholamine that performs the function of a hormone as well as NT. When this NE acts as a stress hormone, it influences the various portions of the brain involved in attention and activities. NE is synthesized by utilizing the enzyme dopamine beta-hydroxylase from DA. It is synthesized inside cells that derive from nuclei of the brainstem, like the locus coeruleus. Nevertheless, NE often has body-wide effects, e.g., throughout the peripheral areas of the nervous system, and is directly released into the bloodstream, through an area known as the adrenal medulla. It is also functioning throughout the peripheral nerves because it triggers the sympathetic ready-to-respond mechanism of the body. NE is the chemical that influences the level of “arousal” in the brain, that is to say, it helps prop up brain systems in preparedness for action [98,99]. Consequently, it has a broadly modulative effect across a wide variety of brain functions such as wakefulness, memory, and mental clarity, allowing the brain to respond efficiently to any difficulties or threats it encounters. NE is closely linked to its hormonal equivalent—epinephrine—which behaves not just as a NT in the brain, but also as an adreno-receptor hormone in the body. It elicits a series of body-wide modifications that together form what is more widely referred to as the “fight or flight” response, meaning that both the body and the brain can cope with any physical or emotional stressors. The brainstem, hypothalamus, and adrenal glands are sites where NE is produced and released into the bloodstream. It raises the level of consciousness and alertness in the brain. This serves to accelerate the body’s functions and is very important in endogenous epinephrine development. Norepinephrine has been used in mood problems like depression and anxiety, in situations where abnormally low concentration were found in the body [100]. On the other hand, an abnormally high concentration of it can result in a disrupted sleep cycle.

Similarly, with epinephrine, NE correspondingly triggers the fight-or-flight response, by directly elevating the heart rate, stimulating the secretion of glucose from energy storage, and increasing blood flow to skeletal muscle. Norepinephrine can equally overwhelm neuroinflammation when it occurs genuinely in the brain from the locus coeruleus (a nucleus in the brain). Comparatively, when norepinephrine is used as a medication, it increases blood pressure by elevating vascular tone via α-adrenergic receptor activation. Research shows that it increases the production of tears, leading to the lubrication of the eyes. It is typically involved in increasing the quantity of blood supplied to the heart. In adipose tissue, it produces physical heat by burning calories [101]. The production of NE is highly reliant on the occurrence of Tyr (an amino acid existing in proteins like meat, nuts, and eggs).

Some dairy products, like cheese, also contain high quantities of Tyr. It is the primary precursor to DA, which is also a precursor of epinephrine and NE. Tyr is also a precursor of Phe. Remarkably, Phe is a central precursor that is altered into NE, Tyr, DA, and epinephrine. One additional amino acid, L-carnitine, supports brain function and serves as a natural antidepressant. It acts through increasing quantities of NE and serotonin [102]. A velvet bean, also known as cowhage (*Mucuna pruriens*), is a herbal medication that encompasses L-dopa, a DA precursor [103].

There are many food sources of NE. These are bananas, beans, legumes, cheese, meats like chicken, chocolate, fish, seafood, meat, and oatmeal. A study showed that banana peels contain substantial concentrations of NE and DA [104]. Similarly, ginsenosides, a natural product, elevates both NE and DA amounts in the brain [105].

### 3.7. Epinephrine

Epinephrine (Epi) is an excitatory NT produced by the chromaffin cells of the adrenal gland. The precursor of the Epi is an enzyme, phenyl ethanolamine-N-methyltransferase, typically existing solely in Epinephrine-secreting neurons. Epi prepares the body for the fight-or-flight response. That means that when a person is highly stimulated (fear, anger, etc.), extra amounts of Epi are released into the bloodstream. This release of Epi increases the heart rate, blood pressure, and glucose production from the liver (glycogenolysis). In this way, the nervous and endocrine systems prepare the body for dangerous and extreme situations by increasing the nutrient supply to the main organs. 

Epi is a particular NT found inside the brain, which aids communication between neurons. However, because Epi is mostly created by the adrenal glands and has roles outside the brain, it can equally function as a hormone. It is involved in numerous functions in the body [106]. The catecholamines have included DA, epinephrine, and NE NTs. These usually depend on the Tyr amino acid for synthesis but may also use phenylalanine. Studies also found that increasing Tyr improved mood and cognitive performance. However, the ability of Tyr to help with psychiatric disorders is limited evidence.

Epi is a next treatment option for acute allergic reactions (anaphylaxis). Epi decreases airway swelling and obstruction and improves blood circulation; blood vessels are restricted, and the heart rate is elevated, increasing the blood flow to the body’s organs. Epi is offered as a prescription in an autoinjector [107]. There is not enough research about the content of Epi in food sources. However, certain foods like coffee, tea, citrus fruits, bananas, chocolate, cocoa, and vanilla can raise Epi levels.

### 3.8. Histamine

Histamine is an excitatory NT released from histaminergic neurons that project from the hypothalamus of mammals. The cell bodies of these neurons are found in the tuberomammillary nucleus (TMN), a part of the posterior hypothalamus. In this area, His neurons form the His network of the brain, which spreads extensively across the brain and involves axonal projections to the cortex, medial forebrain bundle, and beyond. The TMN’s His neurons are active in regulating cycle of sleep-wake and fostering arousal when stimulated. The neuronal firing rate of His neurons in the TMN is closely associated positively with the arousal state of an individual. Such neurons fire quickly during wakefulness periods, fire very slowly over relaxation/tiredness periods, and stop firing entirely in rapid eye movement sleep (REM) and non-rapid eye movement (NREM) sleep [108,109]. It is involved primarily in the inflammatory response, as well as a range of other functions such as vasodilation and regulation of the immune response to foreign bodies. For example, when allergens are introduced into the bloodstream, His assists in the fight against them causing itching of the skin or irritation of the throat, nose, and/or lungs. Therefore, the adequate release of His is essential for maintaining the functions of the brain. Histamine metabolites are raised in the cerebrospinal fluid of people with schizophrenia whereas H1 receptor binding sites efficiency decreased [110]. Brain His is key to motivation and goal-oriented activities [111]. Recent studies have showed that His is implicated in hunger, food anticipatory reactions, and food intake in a differentiated manner, indicating that it may play a significant role in excessive appetites, not just when it comes to nutrition but also differentiated manner in regard to substances abuse. Even so, preclinical studies for the role of the histaminergic system in both rats and mice suggest a possible function for controlling alcohol consumption, as an inhibition of the H3 receptor is involved in the regulation of His and other NTs release. Histamine reduces the consumption of alcohol and participates in many behavioral tasks, such as administering alcohol intake [112]. Histaminergic pathway dysfunctions can also lead to the initiation and progression of multiple sclerosis and its murine model of experimental autoimmune encephalomyelitis, though the function of the various histamine receptors is complicated and still contentious [113].

Histamine occurs naturally in certain foods, and it is equally distributed throughout the body. His development arises when some bacteria or yeasts alter the amino acid histidine into a substance named histamine [114]. His is a particular chemical substance which is released by the immune cells when a hazardous factor is identified by the immune system, leading to an allergic and inflammatory response. When His is secreted, its outcomes are stomach pain and diarrhea, hypotension, mucous expulsion in the nasal routes and gastrointestinal tract, and abundant additional physiologic impacts. His imbalances can lead to all kinds of symptoms like insomnia, emotional tantrums, and changes in behavior. The imbalance may arise when there are inadequate amounts of diamine oxidase (DAO) which is the enzyme responsible for histamine breakdown in the body. Elevated His in its function as a NT may lead to symptoms such as depressed mood, attention and concentration difficulties, fatigue, inadequate sleep or insomnia, headaches, and immune problems. His acts regulate the sleep-wake cycle, through its role as a NT. High His can decrease levels of GABA which may contribute to disruptions in the sleep cycle and increased anxiety. It may also cause the NE and Epi levels to be imbalanced [109].

Similarly, a study that was conducted in rats to prevent the occurrence of myoclonic jerks and the generalized clonic seizure demonstrated that histidine is the precursor of His. His is produced from the amino acid histidine by an enzyme named histidine decarboxylase and is broken down by the joint functioning of enzymes called as His methyltransferase and monoamine oxidase [115]. His is also released from mast cells when some allergic reaction occurs, or there is some tissue injury. 

Previous reports indicated that the dietary intake of precursors of NTs is critical for proper brain functions. Such findings from a study by Yoshikawa et al. suggest that dietary L-histidine is important for maintaining the concentration of brain histamine and anxiety-like behaviors [116]. Hence, the consumption of foods containing L-histidine and histamine can be effective to maintain the amount of histamine in the body for a healthy nervous system.

The most common food which contains histamine in maximum concentration are fermented foods, such as guava, almonds, brinjal, salami, meat products like sausages, pepperoni, and hot dogs, canned meats cheese, beverage, ketchup, fish, kombucha, milk products like yogurt, kefir, sour cream, buttermilk, cottage and ricotta cheese, kimchi, sauerkraut, sour pickles, miso, natto, seafood, pineapple, soya sauce, tamari, coconut, spinach, spoiled food, tea of any sort, tomatoes, vinegar, yeast foodstuffs [117,118]. Some foods are equally called “histamine releasers”. Based on previous studies, it was proposed that foods which contain a low quantity of histamine stimulate the mast cells to release His. These foods primarily are seasonings, liquor, bananas, chocolate, cocoa beans, some fruits, lemon, lime, grapefruit, egg whites, fish, certain legumes, papaya, peanuts, pineapple, meat like pork, shellfish, spices, spinach, strawberries, and tomatoes [119,120]. 

### 3.9. Aspartate

Asp, a naturally occurring and endogenous amino acid which exists in isoforms D-aspartate (D-asp) and L-aspartate (L-asp). D-aspartate is broadly occurring in animal tissues and primarily acts as a NT. Research has established that it plays a significant role in abundant physiological activities, including nutritional, control of reproduction and hormone biology, and neuron defenses. Indeed, numerous studies have observed the controlling influences of D-asp on the nervous, endocrine, and reproductive system [121]. For instance, D-asp supports the manufacturing and release of hormones like glucocorticoids, prolactin, oxytocin, and steroids [122]. Aspartate, as NTs in decreased quantity, can stimulate virtually every neuron in the CNS.

D-aspartate plays a noteworthy part in the endocrine and nervous system to deter neurological diseases and reproductive issues mainly due to its antioxidant characteristics. D-asp controls nociceptive explicit neuronal electrophysiological action and performance to reflect the pain situations [123]. D-aspartate compatibly functions as a pharmacological means in chronic pain to halt headache. Aspartate is found in the aspartic acid form in specific animal and vegetable sources, such as: oysters, luncheon meats, sausage meat, wild game, as well as sprouting seeds, oat flakes, avocado, asparagus, young sugarcane, and molasses from sugar beets [124,125].

D-aspartate can be formed in various body tissues [126]. The precursor of D-asp is D aspartate racemase, which chiefly subsidizes the production of D-asp by altering L-asp to D-asp. Similarly, Mouse glutamic-oxaloacetic transaminase 1-like one has been described to encrypt Aspartate racemase and manufacture considerably D-asp from L-asp in adult neuronal production [127,128]. Synthesized D-asp can be oxidized through D-asp oxidase (DDO), and tissue. D-aspartate amount is principally controlled through D-asp oxidase action. Similarly, aspartate oxidase is the single enzyme recognized to destroy D-asp selectively [129].

## 4. Discussion

NTs are endogenous substances that enable the neurons in the body to interact with each another. They help the brain, through the mechanism of chemical synaptic transmission, to provide a range of functions. These endogenous chemical substances are an important part of maintaining everyday life and work. Chemical synaptic transfer to postsynaptic receptors mainly occurs through the release of the NT from presynaptic neural cells. Different neurological diseases have been observed due to variations in the levels of particular NTs, such as depression, Parkinson’s disorder, Alzheimer’s disease, and schizophrenia [130].

Recently, the metabolic side effects of medications (e.g., modification of orexigenic/anorexigenic signals) and drug-nutraceutical associations provided new perspectives in the understanding of the nutritional neuroscience interdisciplinary approach. Psychobiotics, various animal foods, vegetables, edible plants, roots, and plant extracts could be considered as natural sources of NTs. Nevertheless, there is a need to further explore the impact of NT precursors’ intake through food since there is no comprehensive evidence concerning their bioavailability or medicinal implications is present. The few papers we succeeded in retrieving from our Database Search, deal with the roles of diet in the biosynthesis of NTs and NTs precursors but the highest number of papers are focused on the different roles of dietary habits in pathological circumstances where some NTs are crucial. However, due to space constraints, this discussion has not been addressed in this manuscript.

**Figure 5:**
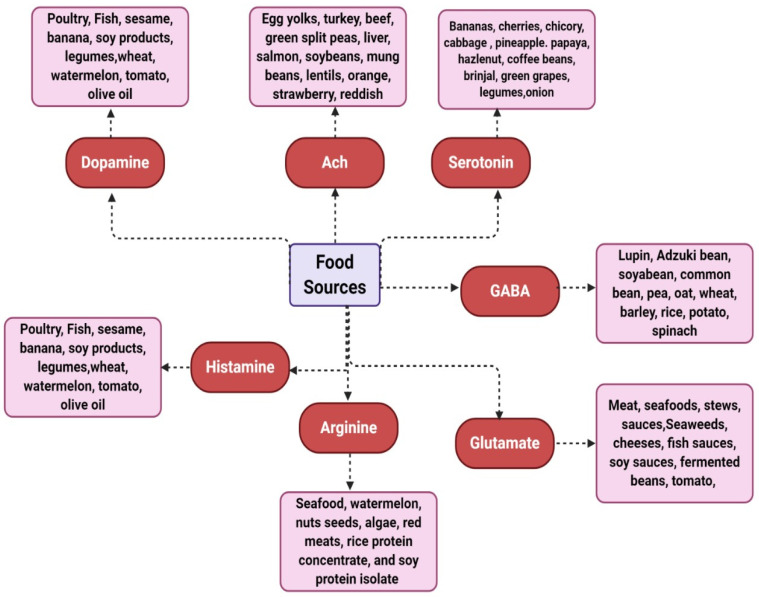
Major NTs and their related food sources.

Figure 5 is representative of the major food sources in which it is possible to get NTs precursors.

Is an adult nervous system unable to manage the homeostatic changes caused by nutritional NT? As research from molecular and neurobiological fields progressively describes the etiology and pathogenesis of neurological disorders, new research studies should explore whether these nutritional NTs could evade the metabolism of the gut microbiota, perform a function on peripheral receptors, being carried across intestinal absorptive cells, bypass the splanchnic metabolism, be transported over blood–brain barriers or impart effects of the central nervous system by circumventricular metabolic processes. In vitro and in vivo studies should be carried out to understand the function of neuronal cells and brain microvasculature to evaluate the potential of nutritional NTs to cross the blood–brain barrier [131]. If promising effects of nutritional NTs on the nervous system are proved through clinical and behavioral studies, the food sources mentioned in Figure 5 would be effective for patients with mental disorders. For example, Alzheimer’s disease or dementia can be controlled via an ACh-rich diet, epilepsy or migraines can be managed through a glutamate-free diet, anxiety, or sleeplessness through the intake of a GABA diet, Parkinson’s disorder by consuming a DA-rich diet, serotonin-rich diet can be effective for depressive disorders and histamine-free diet can manage vascular headaches. Being part of the field of nutritional psychiatry, functional strategies can be applied for either elevated levels of stress or diminished mental outlook disorders [132]. Awareness about these food sources would be a useful starting point for those trying to study their possible impact on mental health, which is a potential threat to many individuals.

## 5. Limitations of the Study and Future Remarks

This article presents an analysis of the biochemical and physiological mechanisms of the influence of NTs on the physical and psychological processes in the human body, as biological and food sources of their synthesis. This review has the main limitation of a scant number of papers dealing with the clinical impact of different dietary habits and folk or traditional cooking methods on NT biology, due to a relative paucity in the literature, despite the huge existence of papers dealing with pathologies involving diets and citing NTs as modulatory elements in the scientific thread of the research description. We have not analyzed specific clinical studies of various diseases associated with NT deficiency, as we intend to do so in future studies. Future remarks should highlight the important role of different food sources as different cultivars in different geographical areas, to assess if the relationship may have epigenetic and genetic foundations. In addition, future studies are needed to investigate the effect of dietary habits on NTs levels and function.

## 6. Conclusions

All the preceding reviewed articles scientifically demonstrated that NTs are essential for the specific activity of body processes, especially the typical processes occurring in the central nervous system. The purpose of this review is to shed light on the fact that imbalance in some major NTs levels are related with severe mental diseases, like, schizophrenia, Parkinson’s disease, depression, and some mood disorders. Similarly, this review article has highlighted some food sources that contain NTs and the positive effects of NTs on the nervous system. Medication can cause some side effects, so nutritional NTs would be useful in such cases. This article focused on the role of plant, vegetable, and animal foods, including dairy products, in order to adequately compensate the body requirement of these NTs. However, there are some foods that should be carefully avoided because they can increase or decrease the release of NTs. Consuming sufficient amounts of basic nutrients that contain precursors for NTs syntheses can be helpful: protein for amino acids, vitamin B, vitamin C, and some oligo-elements may go towards a healthy brain and more balanced NTs levels.

## Data Availability

Not applicable.

## Acronyms

ACh: acetylcholine; Asp: aspartate; DOPA: L-dopamine; Epi: epinephrine; GABA: gamma-aminobutyric acid; Glu: glutamate; His: histamine; NE: norepinephrine; NT: neurotransmitter; Trp: tryptophan; Tyr: tyrosine.
